# Pediatric Sinonasal Rhabdomyosarcoma Presented as Squint: A Case Report and Literature Review

**DOI:** 10.7759/cureus.18548

**Published:** 2021-10-06

**Authors:** Mohamad Shahidatul-Adha, Zubaidah Saizul, Faezahtul-Arbaeyah Hussain, Baharudin Abdullah

**Affiliations:** 1 Department of Ophthalmology & Visual Science, School of Medical Sciences, Universiti Sains Malaysia, Kubang Kerian, MYS; 2 Department of Pathology, School of Medical Sciences, Universiti Sains Malaysia, Kubang Kerian, MYS; 3 Department of Head-Neck Surgery & Otorhinolaryngology, School of Medical Sciences, Universiti Sains Malaysia, Kubang Kerian, MYS

**Keywords:** parameningeal, squint, pediatric tumor, sinonasal tumor, rms, rhabdomyosarcoma

## Abstract

Rhabdomyosarcoma (RMS) is the most common soft-tissue tumor in children, frequently affecting the nose, nasopharynx, and paranasal sinuses. RMS of this sinonasal region manifests with non-specific nasal symptoms of allergic rhinitis, sinusitis, or upper airway infection. Associated ocular symptoms are rare. We describe a young girl who presented with acute onset squint secondary to sinonasal RMS. Imaging showed an aggressive sinonasal tumor with oropharyngeal, intraorbital, and intracranial extension. Histopathological findings were consistent with a diagnosis of rhabdomyosarcoma, embryonal type. The patient deteriorated rapidly due to disease complications. We provide a literature review of pediatric sinonasal RMS with various manifestations.

## Introduction

Rhabdomyosarcoma (RMS) is a malignant tumor featuring skeletal muscle differentiation. It is the most common soft tissue sarcoma among children. However, it is still rare, accounting for less than 5% of all paediatric tumors [1]. Most RMS occur in the head and neck region with three distinct primary tumor sites: orbital, parameningeal and non-parameningeal. Primary orbital RMS is easily diagnosed at an early stage. They are visible with a typical presentation of unilateral rapid evolving proptosis. In contrast, the parameningeal RMS are challenging and difficult to diagnose as it is not visible [2,3]. More than 60% of parameningeal RMS involve paranasal sinuses, while another 30% affect nasal and nasopharynx [3]. Their presenting symptoms are almost similar to allergic rhinosinusitis, which include nasal congestion, mucus discharge, headache, and intermittent nasal bleed. Despite their benign presentation, parameningeal RMS exhibit aggressive behaviour with a high tendency to local and intracranial spread. Herein, we report a case of parameningeal sinonasal RMS in a young girl presenting as squint without anteceding nasal symptoms and provide a literature review on pediatric sinonasal RMS.

## Case presentation

A 4-year-old girl was referred from an otorhinolaryngology (ORL) clinic for eye assessment. She had sudden onset left divergent squint for one month followed by progressive blurry vision and painless proptosis. The right eye developed almost the same symptom two weeks later. The parents reported that the girl had recurrent epistaxis a few days prior to this visit. The patient was otherwise previously healthy and active. There was no history of a blocked nose or rhinorrhea; no diplopia, headache, or vomiting episodes were reported. Past medical history was unremarkable.

Examination of the left eye revealed a marked non-axial proptosis with lagophthalmos, relative afferent pupillary defect, and total ophthalmoplegia. Slit-lamp examination showed a generalised conjunctival injection, exposure keratopathy with inferior paracentral epithelial defect. The right eye had mild proptosis with complete paralysis of abduction; other findings were unremarkable. Fundus examination revealed papilledema with choroidal folds noted on the left eye. Besides ocular findings, the patient also had fullness and swelling of the left cheek. The nose was swollen, slightly distorted with the presence of a hemorrhagic fungating mass in the left nostril. Multiple cervical lymphadenopathies were present. 

**Figure 1:**
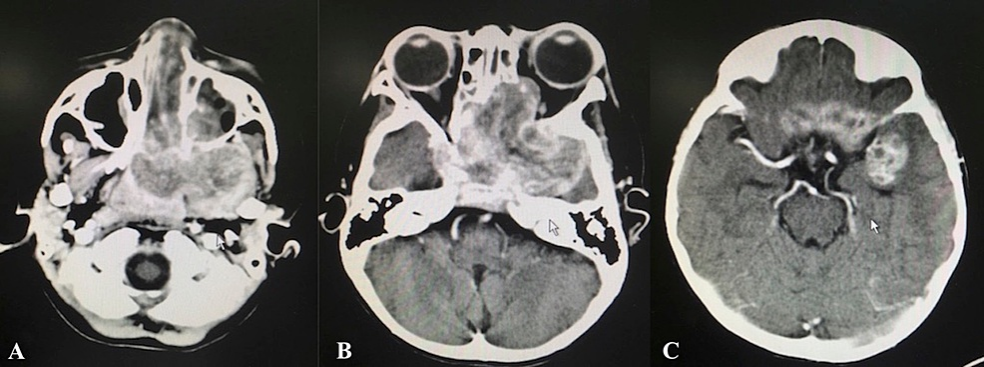
CT scan revealed an extensive sinonasal mass with oropharyngeal (A), intraorbital (B) and intracranial (C) extension.

An urgent computed tomography (CT) scan of the brain, orbit, and paranasal showed an aggressive left sinonasal enhancing mass with intracranial extension superiorly involving bilateral cavernous sinus, bifrontal extradural space, and the left temporal lobe. The tumor also extended to the bilateral orbit, left infratemporal fossa with involvement of left pterygoid muscle, posteriorly occupying the whole nasopharynx and inferiorly to the oropharyngeal region (Figure 1). 

**Figure 2:**
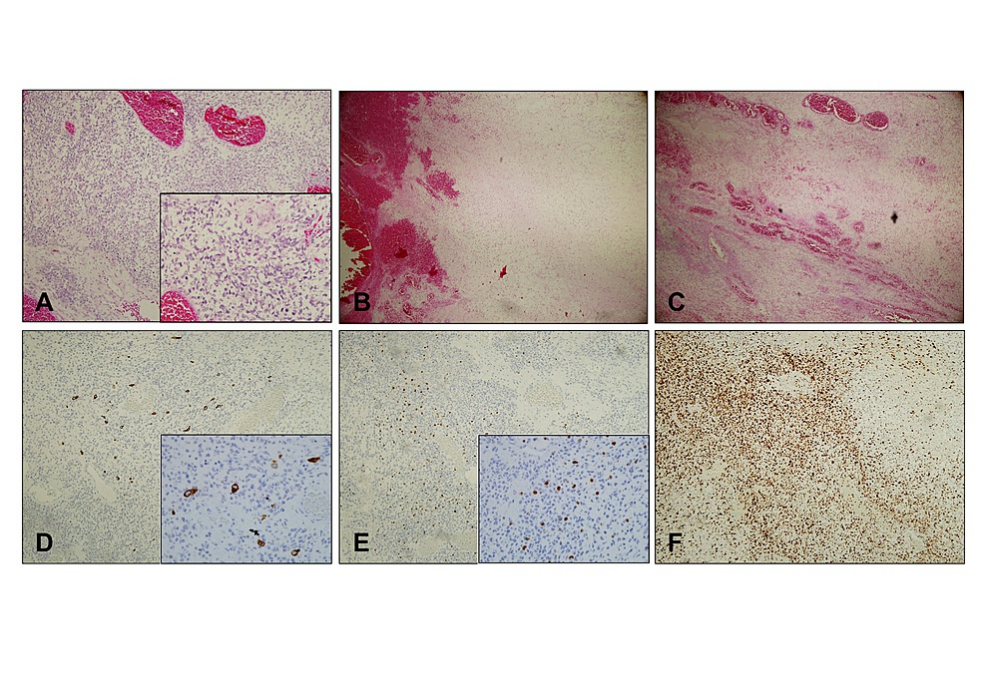
Histological analysis confirmed the diagnosis of embryonal rhabdomyosarcoma. Cytologic section of the nasal tumor mass showed tumour infiltration arranged haphazardly (A, H&E, x100), moderately pleomorphic oval to spindle cells with hyperchromatic nuclei and easily seen mitoses (inlet, x400). A myxoid background in paucicellular region was observed with adjacent haemorrhage (B, H&E, x40) and variable sized blood vessels (C, H&E, x40). Immunohistochemistry study of the tumor cells showed focal positivity to Desmin (D, x100, inlet x400) and Myogenin (E, x100, inlet x400). The tumor cells showed a high proliferative index- Ki 67 of more than 80% (F, x100).

A block of friable nasal mass was detached during a forceful sneezing episode in the ward, prior to the planned biopsy. It was sent for definitive histopathological analysis. The microscopic and immunohistochemical findings were consistent with a diagnosis of rhabdomyosarcoma, embryonal type (Figure 2). 

Both parents were counselled regarding tumor progression and the potential complications. The tumor growth was aggressive and had already advanced to neighboring structures. Thus, tumor radioablation followed with chemotherapy was the best option. However, they decided for alternative traditional treatment. The patient was discharged against medical advice due to parents’reluctance for medical and surgical treatment. 

**Figure 3:**
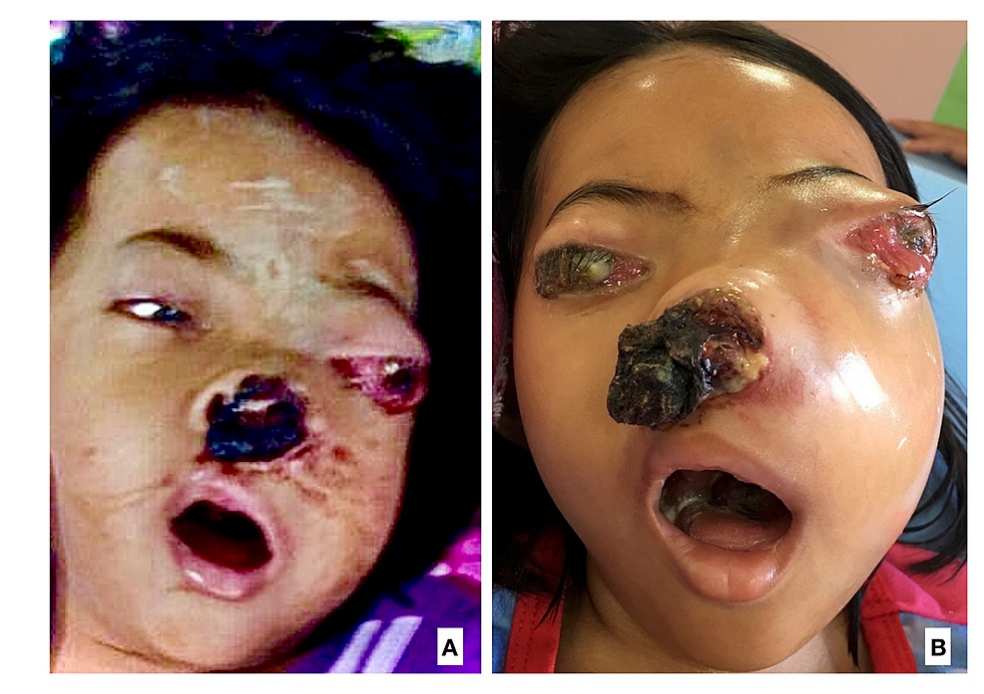
Facial appearance of advanced sinonasal rhabdomyosarcoma in a 4-year-old girl at presentation (A) and one month apart (B). There was a significant growth of the nasal tumor as evidenced by marked generalized swelling of the face and larger necrotic tumor mass from the left nostril distorting the nose. The same characteristic of necrotic tumor mass was also seen within the oral cavity involving the tongue posteriorly, hard palate and posterior buccal mucosa. There was marked proptosis of both eyes with total ophthalmoplegia, with severe exposure keratopathy with ulceration.

About one month after discharge, the parents brought the patient back to the hospital in a severely ill condition. Parents reported that child deteriorated at home, was unable to tolerate neither feeding nor fluids, and was bed-bound ever since. There was severe disfiguration of the face with midfacial fullness, and a huge bulk of necrotic tumor mass protruded out from the nose (Figure 3). The patient succumbed to death on the second day of admission due to tumor progression and complication. 

## Discussion

Sinonasal RMS usually present with symptoms mimicking allergic rhinosinusitis. It is essential to conduct a detailed examination followed with biopsy and histopathological analysis to confirm the diagnosis. A recent epidemiological and end result study has defined six subtypes of RMS, which include embryonal, alveolar, pleomorphic, spindle cell, mixed type, and ganglionic differentiation type [3]. A significant male preponderance was seen in all subtypes [3]. Alveolar and embryonal subtypes had the highest rate of metastasis, yet the 10-year survival rate among embryonal RMS was the highest [3].

We reviewed nine cases of confirmed sinonasal RMS from 2003 until 2017. The pleomorphic subtype was seen only in one case, whilst the rest were embryonal [4-10]. We summarized the findings of the previous cases together with the present case in Table 1. There were three girls and seven boys with the age of presentation between 2-12 years old. Two patients had an ocular complaint as the presenting symptom; one case presented with sudden onset reduced vision, while the other had unilateral protrusion without visual disturbances [4,5].^ ^There was one case in which the parents denied any ocular symptoms but noted the patient to have mild proptosis during the initial assessment [6]. Our patient differs from other cases whereby this patient developed acute onset squint followed by rapid proptosis and blurred vision without anteceding nasal symptoms. The squint seen in our patient is most likely to be a false localising sign, whereby the tumor already invaded the intracranial structure.

**Table 1:** Review cases of pediatric sinonasal rhabdomyosarcoma from 2003 – 2017

Author, Year	Sex/ Age	Tumor location	Presenting symptoms & durations		Histology	Treatment	Clinical course
			Ocular/ Visual	Others			
Parija et al., 2015 [4]	F/12	Left nasopharynx and left posterior nasal cavity.	Sudden blurry vision for 5 days.	Enlarged cervical lymph node, firm, and mobile for 15 days	Embryonal	Chemotherapy VAC with steroid cover.	Remission.
De Melo et.al, 2017 [5]	M/10	Left maxillary sinus, extending to ethmoidal cells, nasal cavity, sphenoid sinus, and left orbit.	Left eye protrusion for 25 days	Left facial swelling for 25 days	Embryonal	Chemotherapy VAC, radiotherapy 50.4 Gy. & surgical ablation	Remission. Vision spared.
Buname et. al, 2014 [6]	M/7	Left nasal cavity, extending to the nasopharynx, maxillary and ethmoidal sinuses, and left orbit	Nil	Bad breath, nasal obstruction, and recurrent epistaxis for 3 months	Embryonal	Debulking, chemotherapy VCD & radiotherapy 50 Gy	Outcome data not available.
Hermann et al, 2003 [7]	M/3	Right nasal cavity, herniating into the right anterior vestibule.	Nil	Unilateral nasal obstruction with purulent rhinorrhea for 3 months.	Botryoid type embryonal RMS	Tumor debulking, chemotherapy VAC.	No recurrence seen on follow-ups.
	M/6	Left middle meatus, extending posteriorly along the lateral nasal wall into the nasopharynx.	Nil	Nasal obstruction 3 months. Left cheek numbness for 2 weeks	Botryoid type embryonal RMS	Tumor debulking, chemotherapy VAC & radiotherapy 4000 cGy	Distant metastasis to lung. Tumor resolution after treatment completion. No recurrence.
	M/11	Right sphenoid sinus, extending into right temporal bone, pterygomaxillary space, and cranial vault with intracranial involvement.	Nil	Frontal headache for 2 months and right facial numbness for 2 weeks	Pleomorphic	Chemotherapy VAC and radiotherapy 5000 cGy	Remission.
Ismi et.al, 2015 [8]	M/3	Right nasal cavity, starting at middle meatus level and fulfilling the nasopharynx.	Nil	Nasal obstruction, hearing loss for 3 months, and sleep apnea for 10 days	Botryoid type embryonal	Endoscopic excision, chemotherapy VAC & radiotherapy	At 2 years – free of disease
Duraes et al, 2005 [9]	M/4	Left maxillary sinus, zygomatic and infraorbitary space including the base of the skull.	Nil	Painless facial swelling for 3 months	Embryonal	Chemotherapy VAC	Succumbed at 16^th^ month.
Bostanci et al, 2015 [10]	F/2	Left maxillary sinus, ethmoidal sinus, and nasopharynx.	Nil	Unilateral nasal obstruction with purulent rhinorrhea for 3 months	Embryonal	Endoscopic resection, chemotherapy VD	Local-regional recurrence. Succumbed at 12^th^ month.
Current study, 2020	F/4	Left nasal cavity with oropharyngeal, bilateral intraorbital and intracranial extension.	Squint & proptosis for one month.	Epistaxis from left nostril for 1 week.	Embryonal	Conservative due to parents’ refusal	Succumbed at 4^th^ weeks.
Abbreviations: F, female; M, male; V, vincristine; A, actinomycin-D; C, cyclophosphamide; D, dactionomycin							

Five of the previously reported cases were successfully treated and survived, whereas another two died despite treatment given due to disease progression and complication. Six cases underwent tumor resection via endoscopic approach, followed by chemoradiotherapy or chemotherapy alone. The patient with pleomorphic subtype who already had intracranial extension successfully went into remission after combination treatment of chemoradiotherapy, without any tumor resection [7]. Chemotherapy alone was also an option, for nasal and nasopharynx localisation, as well as for the extensive inoperable paranasal sinus tumor with intracranial extension [4,9]. Our patient had rapid deterioration mainly due to tumor progression and advanced metastasis to brain, in addition to parents’ refusal for treatment. 

Like other childhood cancers, mortality is the main concern. High-risk groups with poor prognostic factors include children with parameningeal localization, larger tumor size, younger age at presentation, and distant metastasis [11,12]. Children with RMS may survive long-term if the tumor is small and localized, and they are slightly older at presentation [12]. Their life expectancies are short if they present at a late stage, with extensive intracranial extension as in our case. Patients who developed systemic complications secondary to immunosuppression also were unlikely to survive [9,10].

It is crucial for paediatricians, oncologists, and radiologists to work together to decide the best option of treatment. Most important is providing clear advice and continuous support to the parents. Multidisciplinary care and multimodality approach composed of aggressive chemotherapy, radiotherapy, and tumor debulking have shown to induce remission, improve survival, thus reducing morbidity and mortality [13].^ ^Despite being mostly fatal, with great care, RMS is curable. 

## Conclusions

An abrupt onset of squint always require urgent evaluation and prompt referral to ophthalmologists, regardless of the age. It could be the first presenting symptom of a life-threatening condition, such as advanced agressive tumor in our case. The survival rates for patients with embryonal RMS are generally the highest, but tumor size, localisation, grade, and presence of metastatic disease were more predictive of survival. Whether primary or secondary, sinonasal RMS generally has a poorer prognosis due to adjacent parameningeal localisation. 
